# Autophagy, telomerase, and endothelial dysfunction in COVID-19–induced cardiac injury: an evidence-graded genetic and epigenetic synthesis

**DOI:** 10.3389/fcvm.2026.1769828

**Published:** 2026-03-05

**Authors:** HariOm Singh, Gaurav Tripathi, Abdul Arif Khan, Amita Verma, Anchal Singh

**Affiliations:** 1 BSL-4 Laboratory, National Institute of Virology (NIV), Pune, India; 2 Department of Pathology and Laboratory Medicine, Faculty of Medicine, University of Calgary, Calgary, AB, Canada; 3 Department of Microbiology, National Institute of Translational Virology and AIDS Research, Pune, India; 4 Bioorganic and Medicinal Chemistry Research Laboratory, Department of Pharmaceutical Sciences, Sam Higginbottom University of Agriculture, Technology and Sciences, Prayagraj, India; 5 Department of Biochemistry, Institute of Science, Banaras Hindu University, Varanasi, India

**Keywords:** autophagy, cardiac injury, cardiovascular biomarkers, COVID-19, endothelial dysfunction, epigenetic regulation, genetic mechanisms, telomerase

## Abstract

**Background:**

Cardiac injury is a frequent and severe complication of COVID-19, yet the molecular mechanisms driving myocardial involvement remain incompletely understood. Dysregulated autophagy, telomerase/telomere biology, and endothelial dysfunction have emerged as biologically plausible and potentially interconnected contributors to COVID-19-associated cardiac injury.

**Methods:**

We conducted a narrative, evidence-graded review of literature retrieved from PubMed and EMBASE, with Google Scholar used selectively as a supplementary source to capture emerging or cross-disciplinary studies. Eligible studies included human investigations and relevant animal models reporting genetic, epigenetic, or molecular alterations in autophagy, telomerase, or endothelial pathways with cardiovascular relevance. Non-English publications, studies lacking primary data, and reports unrelated to cardiovascular or systemic disease mechanisms were excluded. Evidence was stratified as Level I (direct evidence in COVID-19-associated cardiac injury), Level II (COVID-19 systemic or vascular evidence with plausible cardiac relevance), and Level III (non-COVID cardiovascular or systemic disease; hypothesis-generating).

**Findings:**

Across viral, cardiovascular, and systemic contexts, key candidate genes, including *ATG5, ATG7, Beclin-1, TERT, ICAM1*, and *eNOS***-**emerged as potential mediators of COVID-19–related cardiac injury. While endothelial activation is supported by relatively consistent clinical and molecular evidence, direct cardiac-tissue data linking autophagy and telomerase pathways to COVID-19-associated myocardial injury remain limited. These gaps highlight substantial uncertainty regarding causal mechanisms and inter-individual susceptibility.

**Conclusion:**

Autophagy dysregulation, telomere attrition, and endothelial dysfunction represent convergent and biologically plausible mechanisms contributing to COVID-19–associated cardiac injury; however, current evidence remains largely indirect and derived from systemic or vascular compartments rather than cardiac tissue. Cardiac-specific, longitudinal genetic and epigenetic studies are required before these pathways can be considered for biomarker development or therapeutic targeting.

## 1 Introduction

Cardiac injury ranks among the most frequent and serious complications of coronavirus disease 2019 (COVID-19). Infection with severe acute respiratory syndrome coronavirus 2 (SARS-CoV-2) can precipitate both acute and sustained cardiovascular damage extending beyond the respiratory phase of illness. Clinical manifestations include myocarditis, arrhythmias, heart failure, ischemic events, and microvascular dysfunction. Individuals with pre-existing cardiovascular disease, as well as those who develop acute cardiac injury during COVID-19, are at substantially elevated risk of adverse short- and long-term outcomes.

Endothelial dysfunction has emerged as a central feature of COVID-19 pathophysiology. SARS-CoV-2 disrupts endothelial homeostasis through multiple mechanisms, including reduced nitric oxide bioavailability, oxidative stress, increased vascular permeability, glycocalyx shedding, inflammation, and endothelial-to-mesenchymal transition (EndoMT) (1). These alterations promote microvascular injury, thrombosis, and impaired myocardial perfusion and have been implicated in both acute cardiac injury and post-acute cardiovascular sequelae (2).

Beyond individual molecular pathways, genetic and epigenetic regulation has emerged as a critical determinant of COVID-19 pathogenesis and cardiovascular outcomes. SARS-CoV-2–induced changes in DNA methylation, histone modifications, and microRNA expression can influence inflammatory signaling, endothelial activation, thrombosis, and cellular stress responses. Genetic polymorphisms and epigenetic remodeling of key regulatory pathways may therefore contribute to inter-individual variability in susceptibility to cardiac injury and recovery trajectories following infection.

At the molecular level, autophagy is a fundamental cellular quality-control and recycling process essential for cardiomyocyte survival and endothelial integrity under stress conditions. Dysregulated autophagy has been linked to viral infection, inflammation, and cardiovascular disease. Experimental evidence suggests that SARS-CoV-2 interferes with autophagic flux, potentially amplifying oxidative stress, cytokine signaling, and tissue damage (3).

In parallel, telomere biology reflects cellular replicative capacity and biological aging. Several studies have reported accelerated telomere attrition and epigenetic age acceleration in patients with severe COVID-19 (4), while longer telomere length has been associated with improved clinical outcomes, including reduced need for intensive care and mechanical ventilation (5). However, meta-analyses reveal substantial heterogeneity and inconsistent findings across populations (6), underscoring unresolved uncertainty.

**Figure 1:**
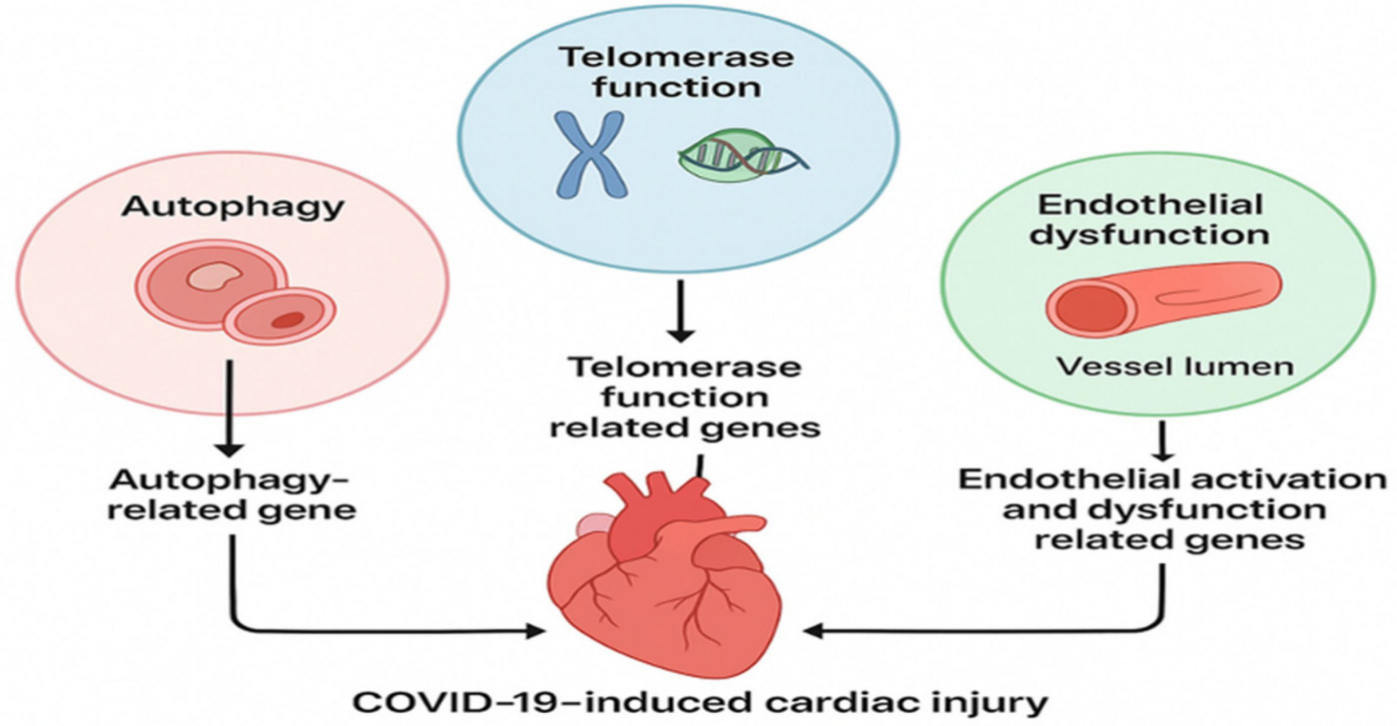
Pathway involved in COVID-19-induced cardiac injury.

Despite growing evidence of endothelial injury and systemic inflammation, the molecular determinants of differential cardiac vulnerability remain poorly understood. Genetic and epigenetic variation in autophagy, telomerase/telomere maintenance, and endothelial function may act as key modifiers of SARS-CoV-2–induced cardiac injury, yet these mechanisms have not been systematically integrated (Figure 1).

The objective of this review is to synthesize and critically evaluate current evidence on genetic and epigenetic alterations in autophagy, telomerase/telomere biology, and endothelial activation in COVID-19–related cardiac injury. Specifically, we aim to (i) map mechanistic links among these pathways, (ii) identify candidate biomarkers of increased cardiovascular risk, and (iii) highlight priorities for future mechanistic and translational research.

Importantly, these pathways are not independent. Oxidative stress and inflammatory signaling induced by SARS-CoV-2 can simultaneously suppress autophagic flux, accelerate telomere shortening, and activate endothelial inflammatory programs. This convergence provides a mechanistic framework linking cellular stress responses to endothelial dysfunction and myocardial vulnerability, potentially explaining inter-individual heterogeneity in COVID-19–related cardiac injury.

## 2 Methodological framework and evidence grading

This review is a narrative, evidence-graded synthesis rather than a systematic review or meta-analysis; therefore, a PRISMA flow diagram was not included. The aim was a critical appraisal and stratification of existing evidence linking autophagy, telomerase biology, and endothelial dysfunction to COVID-19–associated cardiac injury.

Published literature was retrieved from PubMed, EMBASE, and Google Scholar. Inclusion criteria comprised studies on humans or relevant animal models, reporting genetic, epigenetic, or molecular alterations in autophagy, telomerase, or endothelial pathways with potential relevance to cardiac injury. Exclusion criteria included studies lacking primary data, non-English publications, and reports unrelated to cardiovascular or systemic disease mechanisms.

To address heterogeneity, an evidence-grading framework was applied:

Level I: Direct evidence in COVID-19–associated cardiac injury
Level II: COVID-19 systemic or vascular evidence with plausible cardiac relevance
Level III: Non-COVID cardiovascular or systemic disease (hypothesis-generating)

All mechanistic claims and translational interpretations in subsequent sections are explicitly framed according to this evidence-grading hierarchy to avoid over-extrapolation beyond available data.

## 3 Role of autophagy in COVID-19–induced cardiac injury

### 3.1 Autophagy in cardiac homeostasis and viral stress

Autophagy is a conserved lysosomal degradation pathway essential for maintaining cellular homeostasis through the removal of damaged organelles and misfolded proteins. In the cardiovascular system, basal autophagy supports cardiomyocyte survival, limits oxidative stress, and preserves endothelial integrity, particularly under conditions of ischemia, inflammation, and metabolic stress (7, 8).

In the context of viral infection, autophagy serves a dual role by contributing to antiviral defense while also being susceptible to viral subversion. SARS-CoV-2 infection has been associated with myocardial injury manifesting as myocarditis, arrhythmias, heart failure, and microvascular dysfunction (9, 10). Experimental and clinical observations suggest that dysregulated autophagy in cardiomyocytes and endothelial cells may exacerbate inflammation, oxidative stress, and apoptotic signaling, thereby amplifying cardiac injury in COVID-19 (11, 12).

### 3.2 Mechanisms of autophagy dysregulation in COVID-19 cardiac injury

Autophagy is regulated by a coordinated network of autophagy-related genes and signaling pathways, including *ATG5, ATG7, Beclin-1, LC3 (MAP1LC3A)****,*** and the *AMPK–mTOR* axis, which integrates cellular energy status with stress responses (13, 14). In cardiomyocytes, efficient autophagy—particularly mitophagy-is critical for limiting reactive oxygen species (ROS) accumulation and maintaining contractile function. In endothelial cells, autophagy supports barrier integrity and regulates inflammatory activation.

SARS-CoV-2 disrupts autophagy through several convergent mechanisms. Viral proteins have been shown to interact with key autophagy regulators, including Beclin-1 and LC3, impairing autophagosome formation and lysosomal fusion. In parallel, infection-associated metabolic stress promotes mTOR hyperactivation, suppressing autophagic flux and exacerbating cardiomyocyte injury (12). Impaired clearance of damaged mitochondria and protein aggregates results in heightened oxidative stress, inflammatory cytokine release, endothelial dysfunction, and apoptotic cell death. Collectively, these alterations may contribute to myocardial injury, microvascular thrombosis, and fibrotic remodeling observed in severe COVID-19.

**Figure 2:**
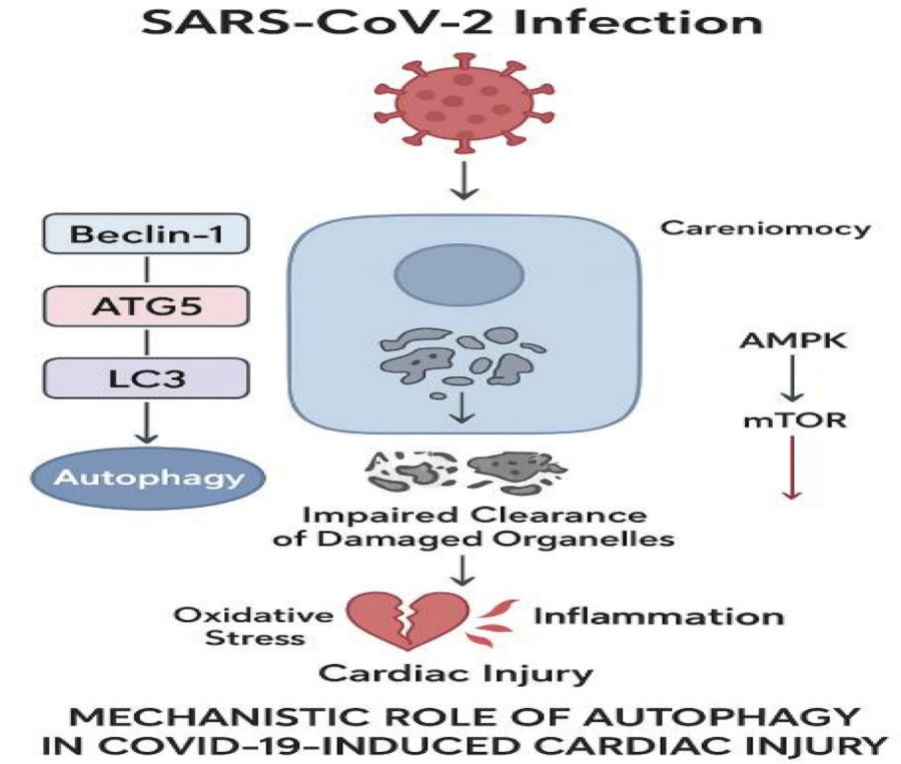
Autophagy dysregulation in COVID-19 cardiac injury.

Notably, impaired autophagy in endothelial cells may exacerbate leukocyte adhesion and microvascular thrombosis, whereas defective mitophagy in cardiomyocytes promotes ROS accumulation and contractile dysfunction, highlighting cell-type–specific consequences of autophagy dysregulation. Collectively, dysregulation of core autophagy mediators such as ATG5, ATG7, and Beclin-1 represents a critical upstream event linking SARS-CoV-2 infection to oxidative stress, endothelial dysfunction, and myocardial injury. The molecular mechanisms underlying SARS-CoV-2–mediated disruption of autophagic flux in cardiomyocytes and endothelial cells are illustrated in Figure 2.

### 3.3 Genetic and epigenetic regulation of autophagy pathways

Genetic variation in autophagy-related genes has been associated with cardiometabolic traits and cardiovascular disease susceptibility in non-COVID contexts, suggesting a potential role as modifiers of COVID-19–related cardiac injury. Variants in *ATG7, ATG5, ATG4C/D, AMBRA1, ATG13, ATG16L1, and MAP1LC3A* have been linked to coronary artery disease, lipid metabolism, insulin signaling, and blood pressure regulation (15). However, direct evidence connecting these variants to myocardial injury in COVID-19 remains limited.

Epigenetic mechanisms, including DNA methylation, histone modifications, and microRNA-mediated regulation, further influence autophagy gene expression and stress responsiveness. SARS-CoV-2–induced inflammatory and oxidative environments may alter epigenetic control of autophagy pathways, thereby contributing to inter-individual variability in cardiac injury severity (Tables 1–3).

**Table 1:** Summary of Genetic and Epigenetic Expression Studies in Cardiac Injuries Induced by COVID-19 and Other Diseases.

S. No.	Candidate gene (Chromosome)	Function	Reports on genetic/epigenetic expression in other diseases	Reports in COVID-19–induced cardiac injury
Autophagy Pathway				
1	ATG5 (6q21)	Essential for autophagy; regulates elongation of autophagic vesicles	↓ Expression with sepsis severity (30); downregulated in coronary artery disease (31)	No report
2	ATG7 (3p25.3)	Activates ATG12 and LC3 during autophagosome formation	Consistent methylation/expression in monocytes of congenital heart disease patients (32)	No report
3	Beclin-1 (17q21.31)	Initiates autophagy via VPS34 complex	↑ Expression in COVID-19 patients (33)	No direct cardiac data
4	AMPK–mTOR (5p13.1, 1p36.2)	Regulates energy balance, growth, and metabolism	Metformin reduced mTOR expression; reversed by AMPK inhibitor (34)	No report
Telomerase Function and Regulation				
5	TERT (5p15.33)	Catalytic subunit of telomerase; maintains telomere length	↓ Expression in hyperoxia-induced mitochondrial injury (35)	No report
6	TERC (3q26.2)	RNA template component of telomerase	Dysregulated; elevated in plasma of HCC patients (36)	No report
7	DKC1 (Xq28)	Stabilizes TERC; essential for telomerase activity	Hub gene in obesity-induced cardiac injury (37)	No report
8	NOP10 (15q14)	snoRNP component involved in RNA pseudouridylation	↑ Expression linked to poor prognosis in NSCLC (38)	No report
9	NHP2 (5q35.3)	snoRNP component; stabilizes telomerase RNA	Mutations reduce telomere length in chronic interstitial lung disease (39)	No report
10	POT1 (7q31.33)	Protects telomere ends; regulates telomerase access	↓ mRNA in malignant vs. benign breast tissues (40)	No report
11	TRF1 (8q13)	Binds telomeric DNA; regulates telomere length	↑ Expression in prostate cancer vs. BPH (41)	No report
12	TRF2 (16q22.1)	Maintains telomere integrity; prevents fusion	↓ Expression causes CD4T-cell dysfunction in chronic viral infection (42)	No report
13	TIN2 (16q12.2)	Links TRF1/TRF2–TPP1; stabilizes telomerase complex	↑ Expression in adult T-cell leukemia (43)	No report
14	TPP1 (16p13.3)	Recruits telomerase to telomeres	Overexpression in aged MSCs improves cardiac function (44)	No report
15	RAP1 (19p13.3)	Associates with TRF2; regulates telomere length	↓ TRF2/RAP1 mRNA in colitis and Crohn's disease (45)	No report
Endothelial Activation and Dysfunction				
16	ICAM1 (19p13.2)	Mediates leukocyte–endothelium adhesion	↑ in atrial fibrillation (46)	No report
17	eNOS (7q36.1)	Synthesizes nitric oxide; regulates vascular tone	↓ Expression in myocardial infarction (47)	No report
18	VCAM1 (1p21.2)	Endothelial adhesion molecule; inflammation mediator	↑ in atrial fibrillation (46)	No report
19	E-selectin (1q24.2)	Mediates leukocyte rolling and adhesion	↑ Expression in atherosclerotic lesions (48)	No report
20	Endocan (5q11.2)	Regulates endothelial inflammation and angiogenesis	Overexpressed in NSCLC (49)	No report
21	IL-6 (7p15.3)	Pro-inflammatory cytokine; immune mediator	↑ in stroke-induced inflammation; IL-6/IL-6R signaling associated with cardiometabolic risk (50, 51)	No report
22	TNF-α (6p21.33)	Pro-inflammatory cytokine; regulates apoptosis	Elevated in ischemia–reperfusion injury, myocarditis, heart failure (52)	No report
23	CRP (1q23.2)	Acute-phase reactant; marker of inflammation	↑ Serum levels in coronary artery disease (53)	No report
24	MMP-7 (11q22.3)	Matrix remodeling and inflammatory mediator	↑ Expression and activity in hypertensive cardiac disease (54)	No report
25	MMP-9 (20q13.12)	Matrix degradation enzyme	↑ Levels in thoracic aortic aneurysm (55)	No report

**Table 2:** Key Genes and Molecular Pathways Implicated in COVID-19–Induced Cardiac Injury.

Pathway	Representative Genes	Molecular Function/Mechanistic Role	Reported Alteration or Association in COVID-19 or CVD	Potential Biomarker/Therapeutic Relevance
Autophagy	ATG5, ATG7, Beclin-1, ATG4C/D, AMBRA1, ATG13, ATG16L1, MAP1LC3A	Regulate autophagosome initiation, elongation, degradation; maintain cardiomyocyte and endothelial homeostasis via AMPK–mTOR signaling	SARS-CoV-2 proteins interact with Beclin-1 and LC3, inhibiting autophagic flux; hyperactivation of mTOR impairs autophagy, increases ROS and apoptosis	Expression/methylation status may indicate risk of myocardial injury; AMPK activators or mTOR inhibitors could restore autophagic balance
	AMPK, mTOR	Energy-sensing regulators of autophagy; coordinate stress and nutrient signaling	mTOR hyperactivation and AMPK inhibition observed in severe COVID-19; linked to metabolic stress and cardiac dysfunction	Pharmacologic modulation (metformin, rapamycin) may mitigate cardiac damage
Telomerase/Telomere Maintenance	TERT, TERC, DKC1, NOP10, NHP2	Components of telomerase complex; maintain telomere length and genomic stability	Decreased telomerase activity and shorter telomeres in COVID-19 patients with severe disease; associated with endothelial senescence and cardiac fibrosis	Telomere length and telomerase activity could serve as biomarkers for cardiac susceptibility; potential for telomerase activation therapies
	Shelterin complex (POT1, TRF1, TRF2, TIN2, TPP1, RAP1)	Protect telomere ends from degradation/fusion; regulate telomerase access	SARS-CoV-2–induced oxidative stress accelerates telomere attrition; downregulation of TRF1/2 linked to senescence pathways	Targets for anti-aging or antioxidant therapies to preserve cardiac integrity post-COVID
Endothelial Activation/Dysfunction	eNOS (NOS3)	Produces nitric oxide for vasodilation and endothelial homeostasis	Reduced eNOS expression and NO bioavailability post–SARS-CoV-2 infection; contributes to endothelial dysfunction and thrombosis	NO donors or eNOS activators may restore vascular function
	ICAM1, VCAM1, E-selectin, Endocan (ESM1)	Mediate leukocyte adhesion and vascular inflammation; markers of endothelial activation	Upregulated during acute COVID-19 and in long-COVID cardiovascular sequelae	Circulating ICAM1/VCAM1 and Endocan as early indicators of endothelial injury
	IL-6, TNF-α, CRP, MMP-7, MMP-9	Inflammatory cytokines and matrix remodeling enzymes	Elevated in severe COVID-19; drive endothelial inflammation, microthrombosis, myocardial remodeling	Candidate serum biomarkers for disease severity and cardiac risk stratification
	Epigenetic Regulators (BRD2, BRD4, DNMT1, SIRT1)	Control endothelial gene expression via chromatin remodeling and DNA methylation	SARS-CoV-2 E-protein interacts with BRD2/4, dysregulating endothelial transcription; DNMT1 and SIRT1 alterations contribute to chronic endothelial dysfunction	BET inhibitors and SIRT1 activators under investigation as endothelial-protective agents

**Table 3:** Epigenetic and miRNA Regulators of Autophagy, Telomerase, and Endothelial Pathways in COVID-19–Induced Cardiac Injury.

Pathway	Regulatory Molecule/miRNA	Target Gene(s)/Function	Observed/Predicted Alteration in COVID-19 or CVD	Functional/Translational Implication
Autophagy	miR-30a, miR-101, miR-376b	Inhibit Beclin-1-mediated autophagy	Up-regulated during viral infection → impaired autophagosome formation	May exacerbate myocardial inflammation; inhibitors could restore autophagy
	miR-21, miR-34a, miR-204	Regulate ATG7, LC3, and AMPK pathways	Elevated in cardiac stress and COVID-19 cytokine storm	Potential plasma biomarkers of autophagy suppression and oxidative stress
	DNMT1, HDAC1/2, SIRT1	Epigenetic control of ATG gene transcription	SARS-CoV-2–induced oxidative stress alters their activity	SIRT1 activation (resveratrol, nicotinamide) may normalize autophagic flux
Telomerase/Telomere Maintenance	miR-138, miR-491, miR-128	Suppress TERT translation	Elevated in inflammatory/viral conditions, reducing telomerase activity	Predictive markers of telomere attrition; therapeutic inhibition may restore telomerase
	miR-200 family, miR-146a	Modulate oxidative-stress and senescence pathways	Dysregulated in aged/post-COVID myocardium	miRNA panels could predict accelerated cardiac aging
	DNMT3A/B, TET2	Regulate TERT and shelterin gene methylation	Aberrant methylation in severe COVID-19	Methylation profiling could stratify cardiac risk
Endothelial Dysfunction	miR-155, miR-126, miR-223	Control ICAM1, VCAM1, and eNOS expression	Dysregulated in COVID-19 plasma and vascular injury	Circulating miR-126 and miR-223 as promising endothelial biomarkers
	BRD2, BRD4 (BET proteins)	Chromatin remodeling at inflammatory gene loci	SARS-CoV-2 E-protein binds BRD2/4 → endothelial gene dysregulation	BET inhibitors (e.g., JQ1) may reduce endothelial inflammation
	SIRT1/H3K9Ac/HDAC9	Maintain endothelial redox balance and NO signaling	Down-regulated in severe cases; hyper-acetylation of ICAM1/VCAM1 promoters	Epigenetic activators (SIRT1 agonists) may restore endothelial homeostasis

Selected single-nucleotide polymorphism (SNP) in autophagy-related genes associated with cardiometabolic and cardiovascular traits are summarized in Supplementary Table S1; however, their relevance to COVID-19–associated cardiac injury remains untested.

### 3.4 Translational implications and future directions

Autophagy-related pathways represent attractive but currently unvalidated targets for mitigating COVID-19–associated cardiac injury. Importantly, pharmacological modulation of autophagy remains experimental, and inappropriate activation or inhibition may be deleterious in the setting of acute viral infection. Therefore, clinical translation should be deferred until cardiac-specific safety and efficacy data are available.

Future research priorities include systematic profiling of autophagy-related genes and epigenetic markers in myocardial tissue, functional studies of SARS-CoV-2 interactions with autophagy machinery, and carefully designed clinical trials evaluating autophagy modulators for cardioprotection.

## 4 Telomerase and telomere biology in COVID-19–induced cardiac injury

### 4.1 Mechanistic role of telomerase

Telomerase is an RNA-dependent DNA polymerase that preserves chromosomal integrity by adding repetitive TTAGGG sequences to telomeres, with TERT encoding the catalytic subunit and TERC serving as the RNA template. While telomerase activity is robust in germline and stem cells, it is low or absent in most somatic tissues, rendering cells susceptible to progressive telomere shortening under conditions of replicative stress, inflammation, and oxidative injury.

SARS-CoV-2 infection induces systemic inflammation, oxidative stress, and excessive reactive oxygen species (ROS) production, all of which accelerate telomere attrition in proliferative cell populations, including leukocytes and endothelial cells (16, 17). Critically short telomeres activate DNA damage response pathways, including p53/p21 signaling, leading to cellular senescence, apoptosis, and the senescence-associated secretory phenotype (SASP), and thereby amplifying inflammation and tissue injury. Epidemiological studies have consistently associated reduced leukocyte telomere length with increased risk of myocardial infarction, stroke, and cardiovascular mortality (18).

**Figure 3:**
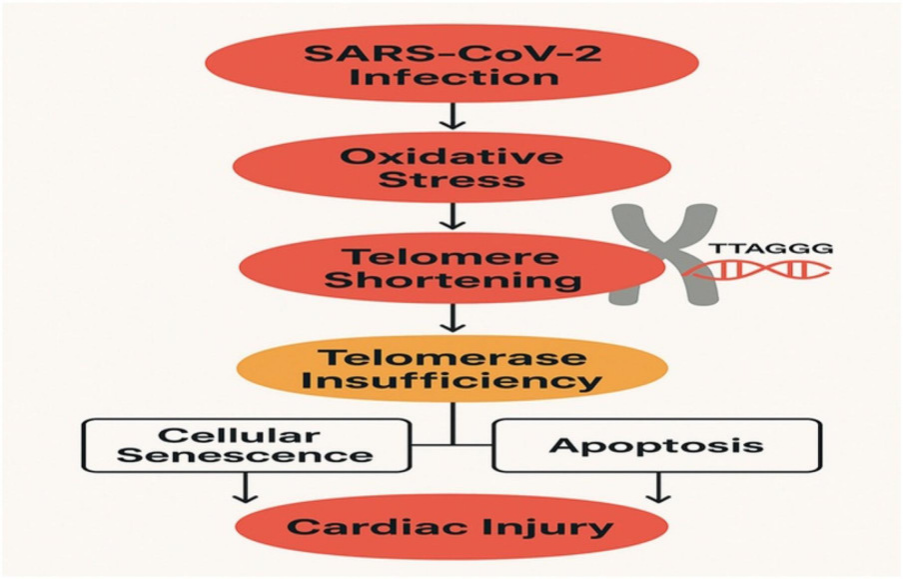
Mechanistic Role of Telomerase in COVID-19–Induced Cardiac Injury SARS-CoV-2 infection may lead to dysregulation of telomerase-associated pathways, primarily inferred from systemic and endothelial evidence, contributing indirectly to cardiac injury, inflammation, and functional deterioration. Key mechanisms include telomere shortening, mitochondrial dysfunction, and increased oxidative stress.

Emerging transcriptomic and epigenetic studies suggest reduced expression of telomerase-related genes (*TERT, TERC, DKC1, NOP10, NHP2****)*** and shelterin complex components **(**POT1, TRF1, TRF2, TIN2, TPP1, RAP1**)** in patients with severe COVID-19 and systemic inflammation (19). Importantly, most available evidence derives from circulating immune cells or systemic tissues rather than myocardial samples (Tables 1–3 and Figure 3). Although telomere shortening is consistently observed in severe COVID-19, direct evidence linking telomerase dysfunction to cardiomyocyte injury remains sparse, and causality cannot currently be inferred. Under sustained inflammatory and oxidative stress conditions, reduced TERT activity may accelerate telomere attrition, thereby promoting endothelial and cardiomyocyte senescence and increasing susceptibility to COVID-19–associated cardiac injury.

### 4.2 Therapeutic considerations and biomarker potential

Potential telomere-preserving strategies—including antioxidants, anti-inflammatory agents, and experimental telomerase activators (e.g., cycloastragenol)—have been proposed to mitigate inflammation-driven telomere erosion. However, these approaches remain hypothesis-generating, and their safety and efficacy in COVID-19-related cardiac injury are unproven. Proposed telomere-preserving strategies should be regarded as hypothesis-generating, as no interventional studies have demonstrated cardioprotective efficacy in COVID-19.

### 4.3 Translational implications

Telomere length and telomerase activity may serve as indirect biomarkers of biological aging and vulnerability to severe COVID-19 outcomes, particularly in older adults and individuals with pre-existing cardiovascular disease. Validation in cardiac-specific, longitudinal cohorts is essential before translational application.

## 5 Endothelial dysfunction in COVID-19–induced cardiac injury

### 5.1 Role of endothelial activation

Endothelial cells regulate vascular tone, growth, thrombogenicity, and inflammation. SARS-CoV-**2** interacts with endothelial cells via ACE2**,** leading to receptor internalization, angiotensin II–mediated vasoconstriction, oxidative stress, and microvascular thrombosis (20, 21).

Endothelial activation is characterized by upregulation of adhesion molecules **(**ICAM1, VCAM1, E-selectin**),** inflammatory mediators **(**IL-6, TNF-α**),** and extracellular matrix–remodeling enzymes **(**MMP-7, MMP-9**),** promoting thrombosis, vascular leakage, and myocardial injury (22, 23). Circulating biomarkers such as endoglin (CD105) and serum amyloid A (SAA) reflect early endothelial stress and vascular inflammation (24, 25). Persistent upregulation of ICAM1 reflects endothelial activation and facilitates leukocyte adhesion, microvascular inflammation, and thrombosis, key pathological features of COVID-19–associated cardiac injury.

### 5.2 Molecular and epigenetic insights

SARS-CoV-2 modulates endothelial gene expression through epigenetic mechanisms. The viral E protein interacts with BRD2/BRD4**,** inducing chromatin remodeling and dysregulated inflammatory gene transcription (26, 27). Epigenetic regulators, including DNMT1/3**,** H3K9 histone modifications, and SIRT1**,** govern adhesion molecule expression, nitric oxide signaling, and endothelial homeostasis. Dysregulation of these mechanisms contributes to sustained endothelial activation and chronic cardiovascular sequelae following COVID-19 (28, 29).

**Figure 4:**
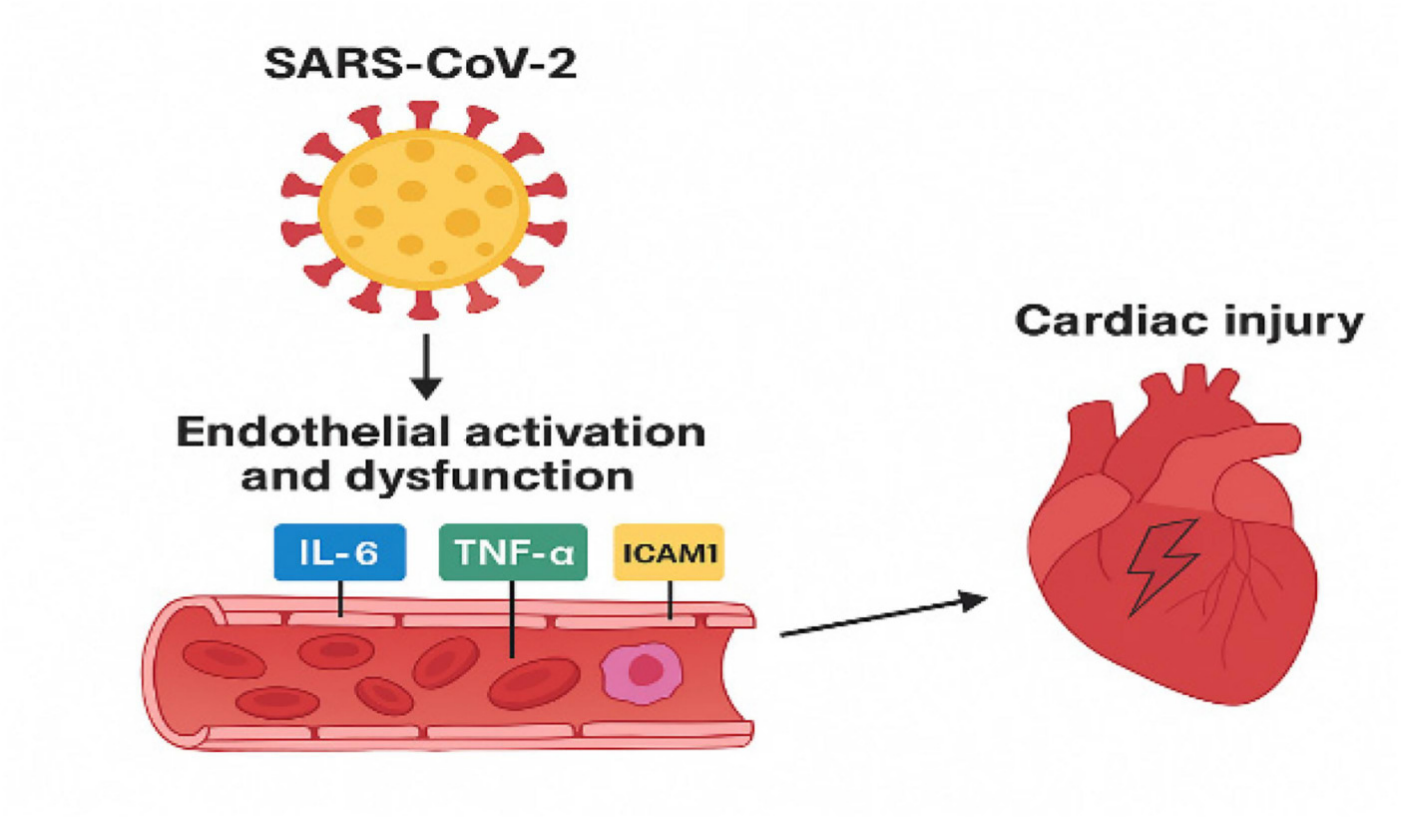
Schematic representation of SARS-CoV-2–induced cardiac injury. Viral infection triggers endothelial activation and dysfunction, leading to elevated levels of pro-inflammatory cytokines (IL-6, TNF-α) and adhesion molecules (ICAM1), which contribute to cardiac damage.

Comprehensive profiling of endothelial markers **(**ACE2, IFN-*γ*, eNOS, VCAM1**)** alongside epigenetic regulators may aid early detection of vascular and cardiac involvement in COVID-19 (Tables 1–3, Figure 4). Through BRD2/BRD4-mediated chromatin remodeling, SARS-CoV-2 may amplify inflammatory endothelial transcriptional programs, thereby indirectly promoting myocardial injury via microvascular dysfunction rather than direct cardiomyocyte infection.

### 5.3 Clinical implications and therapeutic opportunities

High-Risk Populations: Older adults and individuals with pre-existing cardiovascular disease, hypertension, or diabetes are particularly vulnerable to endothelial-mediated cardiac injury.
Biomarker Monitoring: Soluble ICAM1, VCAM1, endocan, SAA, and MMP-9 may facilitate early detection of endothelial activation, pending validation in longitudinal cohorts.
Potential Therapeutics (Experimental): BET inhibitors, ACE2 pathway modulators, antioxidants, and anti-inflammatory agents represent investigational strategies targeting endothelial dysfunction.

### 5.4 Summary and translational relevance

Mechanistic Insights: Endothelial activation and dysfunction drive inflammation, microthrombosis, and myocardial damage in COVID-19.
Genetic/Epigenetic Contributions: BRD2/BRD4, DNMTs, SIRT1**,** and H3K9 modifications modulate endothelial gene expression and stress responses.
Translational Implication: While endothelial dysfunction is strongly supported by vascular and clinical evidence, direct causal links between specific endothelial genetic or epigenetic alterations and myocardial injury remain incompletely established, underscoring the need for cardiac-specific validation studies.

## 6 Cross-Pathway convergence of autophagy, telomerase, and endothelial dysfunction in COVID-19–induced cardiac injury

Rather than acting as independent pathogenic processes, emerging evidence suggests that autophagy dysregulation, telomere/telomerase dysfunction, and endothelial activation form a convergent and interdependent network driving COVID-19–associated cardiovascular injury. This integrative framework represents a shift from descriptive pathway cataloging toward a systems-level model in which disruption of one axis amplifies dysfunction in the others.

Within this model, impaired autophagic flux-mediated by dysregulation of ATG5, ATG7, and Beclin-1-may function as an upstream event that promotes oxidative stress and accelerates telomere attrition. Telomere shortening and reduced TERT activity, in turn, favor endothelial and cardiomyocyte senescence, increasing vulnerability to inflammatory signaling, apoptosis, and microvascular injury. Endothelial activation, reflected by sustained ICAM1 expression, emerges as both a downstream consequence and an amplifying node that reinforces inflammation, thrombosis, and myocardial damage.

**Figure 5:**
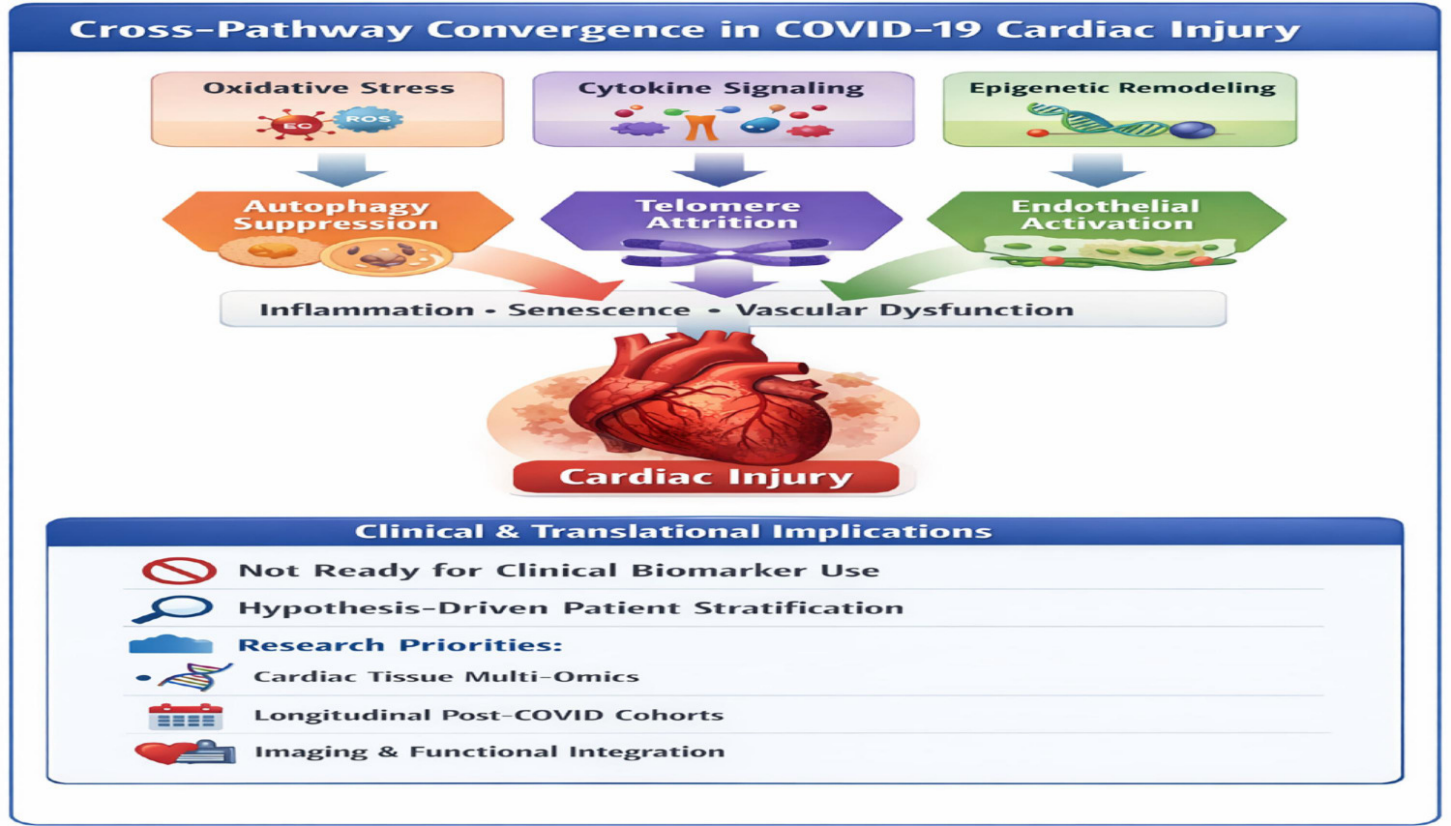
Cross-Pathway convergence in COVID-19 cardiac injury.

Importantly, this convergence model generates testable hypotheses, including the prediction that modulation of autophagy will influence telomere integrity and endothelial function, and that combined assessment of autophagy, telomerase, and endothelial markers will outperform single-pathway indicators in predicting cardiovascular outcomes in COVID-19. Together, these interactions position autophagy–telomere–endothelial cross-talk as a unifying mechanistic axis underlying disease heterogeneity and cardiovascular risk (Figure 5).

## 7 Clinical and translational implications

While these pathways provide mechanistic insight into COVID-19–associated cardiac injury, none currently meet criteria for validated clinical biomarkers or therapeutic targets. As summarized in Table 4, altered autophagic signaling involving Beclin-1, ATG5, ATG7, and LC3-II reflects impaired cellular stress adaptation and may represent modifiable pathways through AMPK–mTOR or SIRT1-targeted interventions aimed at limiting myocardial inflammation and metabolic dysfunction.

**Table 4:** Translational and therapeutic implications of pathway-specific dysregulation in COVID-19–induced cardiac injury.

Pathway	Key Mechanistic Disruption	Potential Diagnostic Biomarkers	Therapeutic/Interventional Strategies	Clinical/Translational Significance
Autophagy	Inhibition of Beclin-1– and ATG-dependent autophagic flux; mTOR hyperactivation.	Circulating ATG5, LC3-II, Beclin-1; plasma miR-30a/-101 levels.	AMPK activators (metformin), mTOR inhibitors (rapamycin), SIRT1 activators.	Restores mitochondrial quality control and limits myocardial inflammation.
Telomerase/Telomere Maintenance	Telomere shortening and reduced TERT/TERC expression → cardiomyocyte senescence.	Leukocyte telomere length, TERT promoter methylation, telomerase activity assays.	Antioxidants (vit C/E, N-acetylcysteine), anti-inflammatories, telomerase activators (TA-65, cycloastragenol).	Biomarkers for biological cardiac aging; may guide risk stratification and anti-senescence therapy.
Endothelial Dysfunction	ACE2 down-regulation, cytokine-induced adhesion molecule expression, NO depletion.	Serum ICAM1, VCAM1, E-selectin, Endocan, IL-6, MMP-9, CRP; miR-126.	ACE2 modulators, NO donors, BET inhibitors, statins, antioxidants.	Early detection of endothelial injury; therapeutic modulation can prevent thrombosis and heart failure.
Cross-Pathway Integration	Interplay among oxidative stress, inflammation, and epigenetic remodeling linking all pathways.	Multi-omics panels combining gene expression, miRNA, and methylation markers.	Precision medicine approach integrating pathway-specific interventions.	Enables comprehensive cardiac risk prediction and targeted post-COVID care.

Markers of telomere attrition and reduced expression of TERT/TERC, primarily derived from circulating or systemic tissues, suggest accelerated cellular aging and senescence in severe COVID-19. While direct cardiomyocyte-specific evidence remains limited, these alterations highlight potential therapeutic avenues involving antioxidant strategies**,** anti-inflammatory modulation**,** or experimental approaches targeting telomere stability.

Endothelial activation and dysfunction, reflected by elevated ICAM1, VCAM1, E-selectin, endocan, IL-6, MMP-9, and CRP**,** represent the most clinically tractable pathway, given the availability of vascular biomarkers and established cardiovascular therapies. Interventions targeting ACE2–angiotensin signaling**,** nitric oxide bioavailability**,** and endothelial inflammation (e.g., statins or anti-inflammatory agents) may offer potential cardioprotective benefits in high-risk COVID-19 populations.

**Figure 6:**
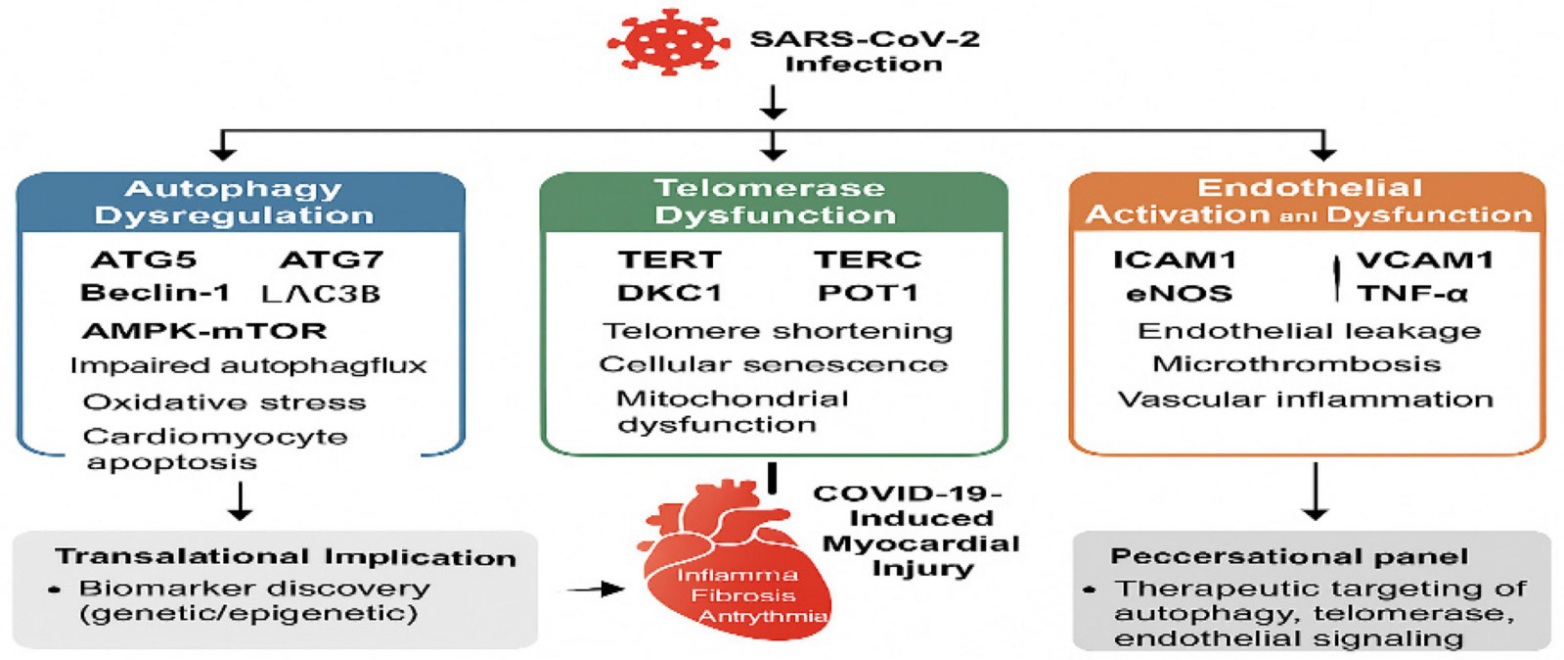
Schematic representation of interconnected autophagy, telomerase, and endothelial dysfunction pathways in COVID-19–induced cardiac injury.

Collectively, cross-pathway convergence driven by oxidative stress, inflammation, and epigenetic remodeling supports the development of integrated multi-omic panels, rather than single biomarkers, for improved risk stratification and precision cardiovascular management **(**Table 4, Figure 6).

## 8 Conclusion and future directions

In conclusion, COVID-19–associated cardiac injury arises from an intricate interplay of cellular stress pathways rather than isolated molecular events. SARS-CoV-2 infection induces oxidative stress and inflammatory signaling that concurrently suppress autophagic flux in cardiomyocytes and endothelial cells, accelerate telomere attrition, and promote endothelial activation. These processes are mechanistically interconnected: impaired autophagy leads to the accumulation of damaged organelles and reactive oxygen species, which in turn exacerbate telomere shortening and endothelial senescence, while telomere dysfunction further increases cellular susceptibility to apoptosis and inflammatory signaling. Endothelial activation, amplified by both autophagy impairment and telomere attrition, contributes to microvascular dysfunction, thrombosis, and subsequent myocardial injury.

This integrated network positions autophagy, telomerase/telomere maintenance, and endothelial function as convergent modifiers of disease severity, providing a unified mechanistic framework to explain inter-individual variability in COVID-19–related cardiac outcomes. Importantly, much of the current evidence remains indirect and is largely derived from systemic or vascular compartments, underscoring the need for cardiac-resolved, longitudinal, multi-omic studies to validate these mechanistic links.

From a translational perspective, this convergence model supports the development of multi-omic biomarker panels integrating autophagy markers (e.g., Beclin-1, ATG5, ATG7), telomere/telomerase indicators (e.g., TERT, TERC, telomere length), and endothelial activation markers (e.g., ICAM1, VCAM1, eNOS) to improve risk stratification and precision cardiovascular management in COVID-19. Future studies should prioritize experimental validation to determine whether targeting one pathway, such as autophagy modulation, can beneficially influence telomere integrity and endothelial function, thereby providing mechanistic and therapeutic insight into this interconnected network.
