# Supplementary material for: Autophagy, telomerase, and endothelial dysfunction in COVID-19–induced cardiac injury: an evidence-graded genetic and epigenetic synthesis
Source: Front Cardiovasc Med. 2026 Mar 5;13:1769828. doi: 10.3389/fcvm.2026.1769828 (PMC12999387; doi:10.3389/fcvm.2026.1769828)
Supplement: Supplementary file 1 [file Supplementaryfile1.pdf]

Supplementary Material

For: Autophagy, Telomerase, and Endothelial Dysfunction in COVID-19–Induced Cardiac Injury: An Evidence-Graded Genetic and Epigenetic Synthesis
Singh HO et al.
Article ID: 1769828

Supplementary Methods

S1. Literature Search Strategy

A structured literature review was conducted using PubMed and EMBASE, with cross-validation through Google Scholar to capture interdisciplinary studies.
Search period: January 2020 – January 2026
Language restriction: English
Study types included: Human studies, experimental models, translational research, mechanistic investigations, genetic and epigenetic analyses.
Search Strings (Representative Examples)

- (“COVID-19” OR “SARS-CoV-2”) AND (“cardiac injury” OR myocarditis OR myocardial OR cardiovascular)
- (“autophagy” OR ATG5 OR ATG7 OR Beclin-1 OR LC3 OR AMPK OR mTOR) AND (“COVID-19”)
- (“telomere” OR “telomerase” OR TERT OR TERC OR shelterin) AND (“COVID-19” OR cardiovascular)
- (“endothelial dysfunction” OR ICAM1 OR VCAM1 OR eNOS OR BRD2 OR BRD4 OR DNMT OR SIRT1) AND COVID-19

Study Selection Process

- Title and abstract screening
- Full-text eligibility assessment
- Manual cross-referencing of bibliographies
- Exclusion of purely descriptive or non-mechanistic commentary

Supplementary Evidence-Grading Framework (Expanded)

Level I: Direct evidence in COVID-19 cardiac tissue (biopsy, autopsy, myocardial profiling)
Level II: COVID-19 systemic/vascular data with strong cardiac biological plausibility
Level III: Non-COVID cardiovascular or systemic mechanistic inference

Supplementary Table S1. Autophagy-Related Genes in COVID-19 Cardiac Injury

Gene	Functional Role	Reported COVID-19 Alteration	Cardiac Relevance	Evidence Level

ATG5	Autophagosome formation	Downregulated in severe disease	Impaired cardiomyocyte autophagy	II
ATG7	Autophagosome elongation	Reduced expression	Mitochondrial dysfunction	II
BECN1	Autophagy initiation	Dysregulated	Viral replication interface	II
MTOR	Autophagy inhibition regulator	Hyperactivated in inflammation	Cardiomyocyte stress signaling	II
AMPK	Energy sensor/autophagy activation	Impaired in metabolic stress	Protective in myocardium	II

Supplementary Table S2. Telomere and Telomerase Pathway Genes

Gene	Function	COVID-19 Association	Cardiac Implication	Evidence Level
TERT	Telomerase catalytic subunit	Reduced activity in severe cases	Cardiomyocyte senescence	II
TERC	Telomerase RNA template	Telomere instability correlation	Myocardial aging vulnerability	III
POT1	Shelterin complex component	Telomere protection	Genomic stability in cardiac cells	III
TRF1/TRF2	Telomere binding proteins	Telomere shortening association	DNA damage signaling	III

Supplementary Table S3. Endothelial Epigenetic Regulators

Gene/Protein	Function	COVID-19 Impact	Cardiovascular Effect	Evidence Level
BRD2/BRD4	Chromatin readers	Viral transcription modulation	Endothelial inflammation	II
DNMT1	DNA methylation	Epigenetic reprogramming	Vascular dysfunction	II

SIRT1	Deacetylase	Downregulated in severe inflammation	Endothelial senescence	II
ICAM1/VCAM1	Adhesion molecules	Upregulated	Microvascular inflammation	I–II

Supplementary Limitations

- Limited availability of cardiac tissue-specific multi-omic datasets in COVID-19.
- Reliance on systemic and vascular extrapolation.
- Absence of prospective longitudinal myocardial profiling.
- Lack of unified integrated genomic–epigenomic cardiac datasets.

Supplementary Future Directions

- Cardiac tissue–specific multi-omics validation.
- Longitudinal telomere-autophagy interaction studies.
- Endothelial chromatin remodeling mapping in COVID-19 myocarditis.
- Integration with clinical severity phenotyping.
